# Contemporary *N*_e_ estimation using temporally spaced data with linked loci

**DOI:** 10.1111/1755-0998.13412

**Published:** 2021-06-22

**Authors:** Tin‐Yu J. Hui, Jon Haël Brenas, Austin Burt

**Affiliations:** ^1^ Department of Life Sciences Silwood Park Campus Imperial College London Ascot UK; ^2^ Big Data Institute Li Ka Shing Centre for Health Information and Discovery University of Oxford Oxford UK; ^3^ Wellcome Sanger Institute Wellcome Trust Genome Campus Saffron Walden UK

**Keywords:** effective population size, linkage disequilibrium, recombination, temporal samples

## Abstract

The contemporary effective population size $N_{e}$ is important in many disciplines including population genetics, conservation science and pest management. One of the most popular methods of estimating this quantity uses temporal changes in allele frequency due to genetic drift. A significant assumption of the existing methods is the independence among loci while constructing confidence intervals (CI), which restricts the types of species or genetic data applicable to the methods. Although genetic linkage does not bias point $N_{e}$ estimates, applying these methods to linked loci can yield unreliable CI that are far too narrow. We extend the current methods to enable the use of many linked loci to produce precise contemporary $N_{e}$ estimates, while preserving the targeted CI width and coverage. This is achieved by deriving the covariance of changes in allele frequency at linked loci in the face of recombination and sampling errors, such that the extra sampling variance due to between‐locus correlation is properly handled. Extensive simulations are used to verify the new method. We apply the method to two temporally spaced genomic data sets of *Anopheles* mosquitoes collected from a cluster of villages in Burkina Faso between 2012 and 2014. With over 33,000 linked loci considered, the $N_{e}$ estimate for *Anopheles coluzzii* is 9,242 (95% CI 5,702–24,282), and for *Anopheles gambiae* it is 4,826 (95% CI 3,602–7,353).

## 1 INTRODUCTION

Effective population size ($N_{e}$) is an important parameter in population genetics. It governs the number of mutants in a population, and hence nucleotide diversity and the number of segregating sites (Charlesworth & Charlesworth, 2010; Wang et al., 2016). It also determines the magnitude of genetic drift, and therefore the stability of allele frequencies over time. Estimates of $N_{e}$ are used in population management, for both conservation and pest control purposes (Lehmann et al., 1998; Waples, 1989). For endangered populations, a certain level of $N_{e}$ needs to be maintained to avoid inbreeding or excessive accumulation of deleterious mutations (Wang et al., 1999). In contrast, if one wishes to reduce the size of a harmful species, $N_{e}$ can act as an indicator to monitor the efficacy of the relevant control measures (Antao et al., 2011). All these applications require robust $N_{e}$ estimates from genetic information. Depending on the questions of interest, some studies estimate $N_{e}$ for over tens or hundreds of thousands of generations, while some, as in this work, focus on a more contemporary time frame, from the most recent to a few generations ago (Waples, 2005).

Linkage disequilibrium (LD) and temporal changes in allele frequency are two established sources of information to estimate contemporary $N_{e}$ (Luikart et al., 2010). The former utilizes LD signal among unlinked and genetically neutral loci, as genetic drift induces nonzero LD (measured by *r* ^2^) under finite $N_{e}$ (Hill, 1981). The reason why unlinked loci are used in the LD method is because they provide information about the $N_{e}$ of the parental generation of the samples but little about the population histories further backward in time (Hayes et al., 2003; Waples, 2005). Drift also causes allele frequencies to fluctuate over generations, and hence $N_{e}$ can be estimated through measuring the temporal changes in allele frequency from a collection of primarily neutral and unlinked loci. Within the temporal methods family there are the moment‐based *F* statistics (Jorde & Ryman, 2007; Krimbas & Tsakas, 1971; Nei & Tajima, 1981; Pollak, 1983; Waples, 1989), while more advanced likelihood methods have also been developed (Hui & Burt, 2015; Wang, 2001; Williamson & Slatkin, 1999). These temporal methods estimate the harmonic mean of the $N_{e}$ between the two (or more) sampling events (Waples, 2005). Additionally, the temporal signals from loci under selection (or linked selection) also hold information about $N_{e}$ (Buffalo & Coop, 2019; Khatri, 2016; Wang et al., 2016).

The existing temporal methods for $N_{e}$ estimation were developed with the use of unlinked loci, and hence only provide methods to calculate confidence intervals (CI) for such data. While applying these methods to linked loci could still provide the same $N_{e}$ point estimate, the inference of CI requires the assumption of independence among loci (Hui & Burt, 2015; Wang, 2001). Clearly this assumption does not hold for linked loci, where temporal changes in allele frequency are correlated. Previous attempts to work around the problem include estimating $N_{e}$ along sliding windows or by resampling of loci (Jónás et al., 2016), but neither of these seems to solve the problem directly or make the best use of the available data. With the advance of sequencing technologies, such as restriction site‐associated DNA (RADseq) and whole‐genome sequencing, obtaining data for large numbers of linked loci is becoming increasingly feasible and affordable. This simultaneously creates the issue of pseudoreplication when computing genomic statistics. This problem arises when loci in tight linkage or proximity provide correlated information (Patterson et al., 2006; Waples et al., 2020). If not handled properly, it will mislead us to overestimate the amount of information contained in the samples. Extending the temporal methods to appropriately incorporate genetic linkage in CI estimation is therefore essential.

## 2 THEORY

We will first derive the covariance of the changes in allele frequency for a pair of linked loci under the Wright–Fisher model, and then find the same covariance under the presence of sampling error. Then, we will use this result to approximate the sampling distribution of the temporal *F* statistic when loci are linked. For tidiness, only key results or equations are displayed in the main text, while the full derivations are given in the Appendix [Link], [Link] for interested readers.

### 2.1 Covariance of the changes in allele frequency between two linked loci

Consider a pair of neutral biallelic loci *i* and *j* with recombination rate *c_ij_*, and let the initial haplotype frequencies be $\underline{p_{ij0}}$. In the next generation, the number of the four haplotypes follow a multinomial distribution with size 2$N_{e}$, and with probabilities equal to the gametic frequencies after recombination. Let *p_it_* and *p_jt_* be the allele frequencies of the first allele at the two loci at generation *t*. The covariance between them is

(1) $$cov\left(p_{\mathrm{it}},p_{\mathrm{jt}}|\underline{p_{ij0}}\right)=\frac{\left(1‐c_{\mathrm{ij}}\right)\left[1‐{\left(1‐\frac{1}{2N_{e}}\right)}^{t}{\left(1‐c_{\mathrm{ij}}\right)}^{t}\right]}{2N_{e}\left[1‐\left(1‐\frac{1}{2N_{e}}\right)\left(1‐c_{\mathrm{ij}}\right)\right]}D_{ij0}$$

where *D_ij_*_0_ is the LD measure between the two loci in the first temporal sample. Waples (1989), Equation 2 derives the variance of allele frequency due to genetic drift, and Equation 1 above can be viewed as an extension of the same formula for the covariance between two allele frequencies.

### 2.2 With sampling error

Usually true frequencies can only be estimated through sampling. Here we consider samples are taken with replacement, or after reproduction. This is equivalent to “sampling plan II” of Waples (1989). The observed haplotype counts can be modelled by another multinomial distribution. Let *x_i_*_0_ and *x_j_*_0_ be the observed frequencies of the first allele at both loci, and *S*_0_ be the diploid sample size (i.e., 2*S*_0_ haplotypes) at generation 0. The covariance between the two observed frequencies is:

(2) $$cov\left(x_{i0},x_{j0}|\underline{p_{ij0}}\right)=\frac{D_{ij0}}{2S_{0}}$$

Similarly, given the true frequencies and diploid sample size *S_t_* at generation *t*, the covariance between the two observed frequencies *x_it_* and *x_jt_* is:

(3) $$cov\left(x_{\mathrm{it}},x_{\mathrm{jt}}|\underline{p_{\mathrm{ijt}}}\right)=\frac{D_{\mathrm{ijt}}}{2S_{t}}$$

By applying the total law of covariance, the covariance of *x_it_* and *x_jt_* given $\underline{p_{ij0}}$.is:

(4) $$\begin{array}{c} cov\left(x_{\mathrm{it}},x_{\mathrm{jt}}|\underline{p_{ij0}}\right)=E\left(cov\left(x_{\mathrm{it}},x_{\mathrm{jt}}|\underline{p_{\mathrm{ijt}}}\right)|\underline{p_{ij0}}\right)\\ \;+cov\left(E\left(x_{\mathrm{it}}|\underline{p_{\mathrm{ijt}}}\right),E\left(x_{\mathrm{jt}}|\underline{p_{\mathrm{ijt}}}\right)|\underline{p_{ij0}}\right)=E\left(\frac{D_{\mathrm{ijt}}}{2S_{t}}|\underline{p_{ij0}}\right)+cov\left(p_{\mathrm{it}},p_{\mathrm{jt}}|\underline{p_{ij0}}\right)\\ \;=\frac{{\left(1‐\frac{1}{2N_{e}}\right)}^{t}{\left(1‐c_{\mathrm{ij}}\right)}^{t}}{2S_{t}}D_{ij0}+\frac{\left(1‐c_{\mathrm{ij}}\right)\left[1‐{\left(1‐\frac{1}{2N_{e}}\right)}^{t}{\left(1‐c_{\mathrm{ij}}\right)}^{t}\right]}{2N_{e}\left[1‐\left(1‐\frac{1}{2N_{e}}\right)\left(1‐c_{\mathrm{ij}}\right)\right]}D_{ij0} \end{array}$$

The first term of Equation 4 follows from Hill and Robertson (1968). The temporal changes in observed frequencies on the two loci are (*x_it_* ‐ *x_i_*_0_) and (*x_jt_* – *x_j_*_0_) respectively. The covariance between them is:

(5) $$\begin{array}{c} cov\left(x_{\mathrm{it}}‐x_{i0},x_{\mathrm{jt}}‐x_{j0}|\underline{p_{ij0}}\right)=cov\left(x_{\mathrm{it}},x_{\mathrm{jt}}|\underline{p_{ij0}}\right)‐cov\left(x_{i0},x_{\mathrm{jt}}|\underline{p_{ij0}}\right)\\ \;‐cov\left(x_{\mathrm{it}},x_{j0}|\underline{p_{ij0}}\right)+cov\left(x_{i0},x_{j0}|\underline{p_{ij0}}\right) \end{array}$$

Because of linearity this covariance can be further broken down into four other covariances. The two terms $cov(x_{i0},x_{\mathrm{jt}}|\underline{p_{ij0}})$ and $cov(x_{\mathrm{it}},x_{j0}|\underline{p_{ij0}})$ are 0 under sampling plan II, as samples are taken after reproduction without affecting the contents of the gamete pool (Waples, 1989). Therefore,

(6) $$\begin{array}{c} cov\left(x_{\mathrm{it}}‐x_{i0},x_{\mathrm{jt}}‐x_{j0}|\underline{p_{ij0}}\right)=cov\left(x_{\mathrm{it}},x_{\mathrm{jt}}|\underline{p_{ij0}}\right)+cov\left(x_{i0},x_{j0}|\underline{p_{ij0}}\right)\\ \;=D_{ij0}\left[\frac{1}{2S_{0}}+\frac{1‐c_{\mathrm{ij}}}{2N_{e}c_{\mathrm{ij}}+1‐c_{\mathrm{ij}}}‐{\left(1‐\frac{1}{2N_{e}}\right)}^{t}{\left(1‐c_{\mathrm{ij}}\right)}^{t}\left(\frac{1‐c_{\mathrm{ij}}}{2N_{e}c_{\mathrm{ij}}+1‐c_{\mathrm{ij}}}‐\frac{1}{2S_{t}}\right)\right] \end{array}$$

which is the sum of the two quantities calculated in Equations 2 and 4. These raw changes in observed frequencies need to be normalized across loci. Krimbas and Tsaka (1971) suggest the following standardization:

(7) $$\delta_{i}=\frac{x_{\mathrm{it}}‐x_{i0}}{\sqrt{x_{i0}\left(1‐x_{i0}\right)}}$$

This standardized change in observed frequency δ*_i_*, like the raw measure, has mean about 0. Hereafter, we will drop the conditional term $\underline{p_{ij0}}$ in our covariances as it becomes cumbersome. Now the covariance between their standardized changes in observed frequency is approximately:

(8) $$cov\left(\delta_{i},\delta_{j}\right)\approx r_{ij0}\left[\frac{1}{2S_{0}}+\frac{1‐c_{\mathrm{ij}}}{2N_{e}c_{\mathrm{ij}}+1‐c_{\mathrm{ij}}}‐{\left(1‐\frac{1}{2N_{e}}\right)}^{t}{\left(1‐c_{\mathrm{ij}}\right)}^{t}\left(\frac{1‐c_{\mathrm{ij}}}{2N_{e}c_{\mathrm{ij}}+1‐c_{\mathrm{ij}}}‐\frac{1}{2S_{t}}\right)\right]$$

where *r_ij_*_0_ is the standardized LD measure between the pair of loci *i* and *j* at generation 0 (i.e., the first temporal sample). Note that this expression is remarkably similar to Equation 6, with *r_ij_*_0_ replacing *D_ij_*_0_ for standardization, based on the ratios of expectations. One can use this formula to find the variance of the standardized change in observed frequency at one locus, by substituting *c_ii_* = 0 and *r_ii_*_0_ = 1, that is, the covariance with itself:

(9) $$var\left(\delta_{i}\right)=cov\left(\delta_{i},\delta_{i}\right)\approx \frac{1}{2S_{0}}+1‐{\left(1‐\frac{1}{2N_{e}}\right)}^{t}\left(1‐\frac{1}{2S_{t}}\right)$$

which yields the same equation as in Waples (1989), Equation 7b. We will revisit this equation later as it holds the key to find the point estimate of $N_{e}$. In addition, we can approximate the correlation between two standardized changes in observed frequency, which is the quotient between Equations 8 and 9:

(10) $$corr\left(\delta_{i},\delta_{j}\right)\approx r_{ij0}\frac{\frac{1}{2S_{0}}+\frac{1‐c_{\mathrm{ij}}}{2N_{e}c_{\mathrm{ij}}+1‐c_{\mathrm{ij}}}‐{\left(1‐\frac{1}{2N_{e}}\right)}^{t}{\left(1‐c_{\mathrm{ij}}\right)}^{t}\left(\frac{1‐c_{\mathrm{ij}}}{2N_{e}c_{\mathrm{ij}}+1‐c_{\mathrm{ij}}}‐\frac{1}{2S_{t}}\right)}{\frac{1}{2S_{0}}+1‐{\left(1‐\frac{1}{2N_{e}}\right)}^{t}\left(1‐\frac{1}{2S_{t}}\right)}$$

### 2.3 Point estimation of *F*, and its distribution

Consider a more general case with *K* loci. Let ${\hat{F}}_{a}$ be the arithmetic average of *K* squared standardized changes in observed frequency (Krimbas & Tsaka, 1971):

(11) $${\hat{F}}_{a}=\frac{1}{K}\sum_{i=1}^{K}\delta_{i}^{2}=\frac{1}{K}\sum_{i=1}^{K}\frac{{\left(x_{\mathrm{it}}‐x_{i0}\right)}^{2}}{x_{i0}\left(1‐x_{i0}\right)}$$

${\hat{F}}_{a}$ is a good estimator for *F*, the variance of the standardized change in observed frequency (Equation 9). Therefore, its expectation is approximately (Waples, 1989):

(12) $$E\left[{\hat{F}}_{a}\right]\approx F=\frac{1}{2S_{0}}+1‐{\left(1‐\frac{1}{2N_{e}}\right)}^{t}\left(1‐\frac{1}{2S_{t}}\right)$$

The point estimate $\hat{N_{e}}$ can be obtained by solving the above equation with known sample sizes and *t*. Waples (1989) provides an approximate solution:

(13) $$\hat{N_{e}}\approx \frac{t}{2\left({\hat{F}}_{a}‐\frac{1}{2S_{o}}‐\frac{1}{2S_{t}}\right)}$$

As mentioned, the expectation (Equation 12) holds regardless of genetic linkage. The main difference is the variance and distribution of ${\hat{F}}_{a}$, and therefore the width of CI, depend heavily on the covariance structure among loci.

Let ${\hat{F}}_{a,\;indep}$ be the ${\hat{F}}_{a}$ computed from *K* independent loci. The classical result suggests $K{\hat{F}}_{a,\;indep}/F$ is approximately $\chi_{K}^{2}$ distributed, where the subscript in $\chi_{K}^{2}$ denotes the degrees of freedom, which is also *K* (Waples, 1989). The 95% CI for *F* can be found using the 2.5‐ and 97.5‐percentile of $\chi_{K}^{2}$:

(14) $$95\%C.I.forF=\left[\frac{K{\hat{F}}_{a,indep}}{\chi_{K,0.975}^{2}},\frac{K{\hat{F}}_{a,indep}}{\chi_{K,0.025}^{2}}\right]$$

Similarly, we denote ${\hat{F}}_{a,\;linked}$ as the ${\hat{F}}_{a}$ statistic calculated from *K* linked loci. The distribution of ${\hat{F}}_{a,\;linked}$ should be more dispersed than that from *K* independent loci. To approximate the distribution of $K{\hat{F}}_{a,\;linked}/F$ we let ***R*** be a *K* by *K* correlation matrix for the standardized changes in observed frequency $\left(\delta_{1},\;\delta_{2},\ldots ,\;\delta_{K}\right)$, whose elements are as described in Equation 10. This correlation matrix ***R***, being symmetrical and positively definite, has real positive eigenvalues $\lambda_{1},\;\lambda_{2},\ldots ,\;\lambda_{K}$. $K{\hat{F}}_{a,\;linked}/F$ is approximately distributed as the sum of *K* independent random variables:

(15) $$\frac{K{\hat{F}}_{a,linked}}{F}\dot{\sim }Q^{2}=Q_{1}^{2}+Q_{2}^{2}+\ldots +Q_{K}^{2}$$

where each $Q_{i}$ is independently and normally distributed with mean 0 and variance $\lambda_{i}$. More details about $Q^{2}$ can be found in the Appendix [Link], [Link]. The closed form of $Q^{2}$ is usually not known, but its values can be conveniently computer‐generated. The CI for *F* with linked loci can then be obtained from the empirical quantiles of $Q^{2}$. For instance,

(16) $$95\%C.I.forF=\left[\frac{K{\hat{F}}_{a,linked}}{Q_{0.975}^{2}},\frac{K{\hat{F}}_{a,linked}}{Q_{0.025}^{2}}\right]$$

For the limiting case of having *K* independent loci, ***R*** is simply an identity matrix, whose eigenvalues are all 1, and thus $Q^{2}$ is reduced back to $\chi_{K}^{2}$.

While ${\hat{F}}_{a}$ is a sum‐of‐ratios statistic (Jorde & Ryman, 2007), we also introduce its ratio‐of‐sums counterpart ${\hat{F}}_{b}$:

(17) $${\hat{F}}_{b}=\frac{\sum_{i=1}^{K}{\left(x_{\mathrm{it}}‐x_{i0}\right)}^{2}}{\sum_{i=1}^{K}x_{i0}\left(1‐x_{i0}\right)}$$

We can view ${\hat{F}}_{b}$ as the weighted average of $\delta_{i}^{2}$, where the weights are $w_{i}=x_{i0}\left(1‐x_{i0}\right)/\sum_{i=1}^{K}x_{i0}\left(1‐x_{i0}\right)$. ${\hat{F}}_{b}$ shares the same expectation with ${\hat{F}}_{a}$ (and hence provides the same $\hat{N_{e}}$) but with a slightly different variance. To find the CI for *F* with ${\hat{F}}_{b}$, let $W^{1/2}$ be a diagonal matrix whose (diagonal) elements are $\sqrt{w_{i}}$. We then compute the eigenvalues of $KW^{1/2}RW^{1/2}$ and use them to generate $Q^{2}$, the approximate distribution of $K{\hat{F}}_{b}/F$. The CI for *F* can be found as described previously (Equation 16).

## 3 SIMULATIONS

To summarize, contemporary $N_{e}$ and its CI can be estimated from linked loci via the following steps:

1. Calculate ${\hat{F}}_{a}$ (Equation 11) or ${\hat{F}}_{b}$ (Equation 17).

2. Find the point estimate of the effective population size $\hat{N_{e}}$ (Equation 13).

3. Estimate $r_{0ij}$ for every pair of loci *i* and *j* (see Appendix [Link], [Link]).

4. Calculate the correlation matrix ***R***, using the estimates above, alongside other known parameters *t*, $S_{0}$, $S_{t}$ and $c_{\mathrm{ij}}$ (Equation 10).

5. Compute the eigenvalues of ***R*** (or $KW^{1/2}RW^{1/2}$ if ${\hat{F}}_{b}$ is used).

6. Generate the empirical distribution of $Q^{2}$ from the eigenvalues obtained above. Find the 95% CI for *F* (Equation 16), and convert them into the corresponding upper and lower bound for $N_{e}$ (Equation 13).

Computer simulations were run (see Methods below) to verify the theories behind the two *F* statistics. The main results are shown in Table 1, with additional results in the Appendix [Link], [Link]. The average ${\hat{F}}_{a}$ and ${\hat{F}}_{b}$ followed the expectations very closely in all simulation settings, with only a few per cent deviation. However, the bias was exaggerated in the $N_{e}$ scale in some cases, particularly when the sample size to $N_{e}$ ratio was small. The ratio‐of‐sums ${\hat{F}}_{b}$ performed better under such scenarios with much lower $N_{e}$ bias. The standard deviations of the two estimators were comparable, although those for ${\hat{F}}_{a}$ were consistently slightly smaller. Perhaps the more important results are the width and coverage of the 95% CI. Those inferred from the adjusted $Q^{2}$ distribution covered the true *F* in about 95% of the simulations, which accurately reflected the desired confidence level. The adjusted CI worked for both ${\hat{F}}_{a}$ and ${\hat{F}}_{b}$ statistics, and for both phased and unphased data. For comparison, the last column of Table 1 shows the CI coverage calculated as if loci were independent. These $\chi^{2}$‐based CI coverages were all below the targeted level, and in some cases the coverage was as low as 53%. Additional simulations were run to confirm the method's robustness towards different levels of genome‐wide recombination (see Methods and Appendix [Link], [Link]), and with more linked loci. In short, the adjusted CI remained accurate with the desired coverage under all the scenarios examined.

**TABLE 1:** Simulation results

$N_{e}$	K	$S_{0},\;S_{t}$	t	True F	$Mean\;\left({\hat{F}}_{a}\right)$ & corresponding $\hat{N_{e}}$	$SD\;({\hat{F}}_{a})$	${\hat{F}}_{a}$ adjusted 95% C.I. coverage (phased data)	${\hat{F}}_{a}$ adjusted 95% C.I. coverage (unphased data)	$Mean\;\left({\hat{F}}_{b}\right)$ & corresponding $\hat{N_{e}}$	$SD\;({\hat{F}}_{b})$	${\hat{F}}_{b}$ adjusted 95% C.I. coverage (phased data)	$\hat{F_{b}}$ adjusted 95% C.I. coverage (unphased data)	Unadjusted 95% C.I. coverage (assumed independence)
1,000	500	50, 50	10	0.02494	0.02404 (1,238)	0.00224	0.948	0.947	0.02417 (1,119)	0.00246	0.956	0.952	0.723
1,000	500	100, 100	10	0.01496	0.01441 (1,134)	0.00149	0.955	0.951	0.01457 (1,094)	0.00154	0.958	0.946	0.747
5,000	1,000	100, 100	10	0.01099	0.01064 (7,812)	0.00062	0.966	0.960	0.01078 (6,410)	0.00064	0.960	0.955	0.809
10,000	2,000	100, 100	10	0.01050	0.01014 (35,714)	0.00044	0.970	0.966	0.01029 (17,241)	0.00046	0.962	0.958	0.720
10,000	5,000	100, 100	10	0.01050	0.01015 (33,333)	0.00035	0.960	0.953	0.01030 (16,667)	0.00036	0.969	0.960	0.531

Note

Simulation parameters are given in the first four columns: true $N_{e}$, K, the two sample sizes $\left(S_{0},S_{t}\right)$, and the number of generations between samples t. Recombination frequency was 1e‐5 between adjacent bp per generation, and the chromosome length was 1e5 bp. True F (fifth column) is calculated via equation 12 given the parameters. For each combination of parameters the average of ${\hat{F}}_{a}$ from 1,000 independent simulation was reported in the sixth column, with the corresponding $\hat{N_{e}}$ in parentheses (calculated via equation 13). The standard deviations are shown in the seventh column. The next two columns present the proportions of runs (out of 1,000) in which the 95% C.I. cover the true F, using the adjusted $Q^{2}$ distributions. Phased and unphased data are treated separately (see SI). The next four columns show the same information for ${\hat{F}}_{b}$, the ratio‐of‐sum statistic, using the same simulated datasets. For comparison, the coverage of the unadjusted $\chi_{K}^{2}$ C.I. (i.e. assuming independence, Equation 14) is shown in the last column.

## 4 APPLICATION

**FIGURE 1:**
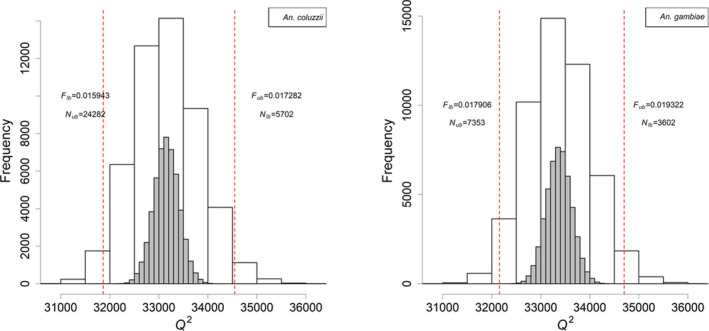
The empirical distributions of $Q^{2}=K{\hat{F}}_{a}/F$ for *Anopheles coluzzii* (left) and *Anopheles gambiae* (right), where K is the total number of linked loci used on both arms of chromosome 3 (33,148 for *A*. *coluzzii*, 33,372 for *A*.* gambiae*). The histograms in white are 50,000 realizations of $Q^{2}$ based on the eigenvalues of ***R***. The red dotted lines mark the 2.5‐ and 97.5‐percentiles of $Q^{2}$, which are the lower and upper confidence interval for *F*. The associated upper and lower 95% CI $N_{e}$ are shown in Table 2 (last column). For comparison, the distributions of $\chi_{K}^{2}$ (i.e., assuming the loci are independent) are shown on the same plots in grey.

We applied the method to two sets of *Anopheles gambiae* and *Anopheles coluzzii* mosquito sequences collected by The *Anopheles gambiae* 1000 Genome Consortium (Ag1000G) in southwest Burkina Faso (Ag1000G, 2017; Ag1000G, 2017). Samples were collected in 2012 and 2014, corresponding to an estimated temporal separation of 20 generations (O'Loughlin et al., 2016). Full details of the data processing and calculation are given in the Methods and Appendix [Link], [Link]. With over 33,000 linked loci considered on chromosome arms 3R and 3L combined, the estimated harmonic mean $N_{e}$ in this period via ${\hat{F}}_{a}$ were 9,242 (95% CI 5,702–24,282) for *A*. *coluzzii*, and 4,826 (95% CI 3,602–7,353) for *A*. *gambiae* (Table 2). The empirical distributions of $Q^{2}$ for the two estimates are shown in Figure 1. Analyses with ${\hat{F}}_{b}$ gave very similar results (Appendix [Link], [Link]).

**TABLE 2:** Summary of $N_{e}$ estimates of *Anopheles coluzzii* and *Anopheles gambiae* from villages in southwest Burkina Faso

Species	Chrom	$S_{0}$	$S_{t}$	K	${\hat{F}}_{a}$	$\hat{N_{e}}$	Combined $\hat{N_{e}}$
*A. coluzzii*	3R	82	53	17,837	0.0166038	9,325 [5,314–36,748]	9,242 [5,702–24,282]
*A. coluzzii*	3L	82	53	15,317	0.0166246	9,148 [5,042–46,489]	
*A. gambiae*	3R	92	45	17,963	0.0184214	5,332 [3,643–9,913]	4,826 [3,602–7,353]
*A. gambiae*	3L	92	45	15,409	0.0188472	4345 [3,051–7,341]	

Note

One set of ${\hat{F}}_{a}$ and $\hat{N_{e}}$ were estimated per chromosome arm (Chrom) initially. For each species two samples with diploid sample sizes $S_{0}$ and $S_{t}$ were collected 2 years apart (assumed to correspond to *t* = 20 generations). Pairwise $c_{\mathrm{ij}}$ were calculated from physical distances via Haldane's mapping function, using the published recombination frequency (1.4 centimorgan per megabase; Pombi et al., 2006). The 95% CIs for $N_{e}$ (given in square brackets) were calculated from the adjusted $Q^{2}$, with 50,000 realizations. The last column combines genotypic information from both chromosome arms to provide an overall $N_{e}$estimate for each species. The same table calculated with ${\hat{F}}_{b}$ can be found in the Appendix [Link], [Link].

## 5 DISCUSSION

Gathering genetic data at two or more time points from a single population ought to be useful for estimating $N_{e}$, but thus far there have been no methods to estimate proper CI when linked loci are used. The existing methods implied or assumed independence among loci when inferring CI, as this was the basis for aggregating information across loci (Wang, 2001; Waples, 1989). This assumption severely limits the species applicable to the methods, or the number of loci to be included in one analysis. For instance, existing temporal methods would have difficulties in estimating the CI of our two examples *Anopheles gambiae* and *Anopheles coluzzii*, which have only two pairs of autosomes and hence very few truly unlinked loci. On the one hand, if genetic linkage is ignored, then the existing $\chi^{2}$ based CI will be too narrow, as demonstrated in our simulations and Figure 1. On the other hand, if only a handful of unlinked loci are selected for $N_{e}$ estimation, then the estimation error will become too large and can often lead to the unwelcome consequences of having infinite $\hat{N_{e}}$ or upper CI (Waples, 1989).

For independent loci, computing $var(\delta_{i})$ alone is sufficient to obtain the point and CI estimates (Waples, 1989). For linked loci, however, the now nonzero between‐locus covariance $cov(\delta_{i},\delta_{j})$ needs to be considered as drift trajectories are correlated. This work calculates this covariance (Equation 8) from the discrete two‐locus two‐allele Wright–Fisher model with recombination, and then uses it to provide a method for finding the appropriate CI. The matrix ***R*** describes the correlation among $\delta_{i}$. We can decorrelate them through eigen‐decomposition of ***R***. Its eigenvalues are crucial in approximating the sampling distribution of $K{\hat{F}}_{a}/F$, and eventually the CI for *F* and $N_{e}$. In principle, this eigen‐decomposition framework can also help tackle the issue of pseudoreplication on other genomic statistics. One potential example is $F_{\text{ST}}$ for population differentiation (Waples et al., 2020). Each locus contributes to the overall $F_{\text{ST}}$ but provides duplicated information. Hence $var({\hat{F}}_{\text{ST}})$ depends on the correlation structure among the individual $F_{\text{ST}}$ across loci, which may also depend on parameters such as $N_{e}$, migration, pairwise LD and recombination rates.

A key component of ***R*** is the initial pairwise LD, measured by $r_{ij0}$. During our development we found that using the maximum likelihood estimate (MLE) for $r_{ij0}$ tends to over‐estimate its magnitude and thus the eigenvalues of ***R***, resulting in CIs being slightly more conservative. This is because finite sampling itself also induces some LD (Hill, 1981). Since an unbiased estimator for $r_{ij0}$ is not found, empirical corrections are imposed, with slightly different treatments for phased and unphased data (see Appendix [Link], [Link]). Another parameter is the pairwise recombination rates $c_{i,j}$, and a fine‐scaled recombination map will be helpful in determining such rates. While a recombination map is not yet available for *A*. *gambiae*, we used a published genome‐wide average recombination frequency (Pombi et al., 2006) and the Haldane mapping function to convert pairwise physical distances into $c_{i,j}$. Further theoretical work would be needed to investigate the consequences of mis‐specifying these rates. While the underlying $N_{e}$ influences the width of CI through affecting the magnitude of average $r_{ij0}$ (Hill, 1981) and pairwise $corr(\delta_{i},\delta_{j})$, we found that ***R*** and the CI are relatively insensitive to mis‐specification of $\hat{N_{e}}$. A 10‐fold over‐ or under‐estimation of $N_{e}$ does not greatly affect the estimated distribution of $\hat{F}$ (see Appendix [Link], [Link]). Better estimates of ***R*** and its components are welcome.

The previous requirement of independent loci precluded the use of high‐throughput sequencing technologies, which potentially yield tens of thousands of linked loci. There have been attempts to estimate $N_{e}$ trajectories from whole‐genome sequencing data, such as by studying the LD decay curves (Hayes et al., 2003) or identity‐by‐descent (IBD) tracks (Browning and Browning, 2015), but these one‐sample methods focus on a much longer horizon (up to thousands of generations) backward in the past. It is also observed that they tend to produce confounded or correlated $N_{e}$ estimates at different time points. Complementary to these works, the temporal method presented here provides an $N_{e}$ estimate that specifically pertains to the time window between samples, without the interference of population size dynamics further in the past (Waples, 2005). It also probes the question of how much genetic information is allocated into the inference of contemporary $N_{e}$. The LD method suggests that loci with recombination rate *c* apart contain information about $N_{e}$ of 1/(2*c*) generations ago (Hayes et al., 2003), which means many loci with shorter distances are not contributing (e.g., *c*>0.025 is required to infer the most recent 20 generations of $N_{e}$). There is a similar claim on the relationship of *c* and timescale for the IBD method. Our proposed method, in contrast, uses loci with any recombination distances to isolate the drift signal between the sampling events. This temporal method also has the potential to work with RADseq data, where linked loci are discovered from multiple RAD fragments of short length (and hence no IBD information). It is a more accessible alternative to whole‐genome sequencing, particularly for nonmodel species, but still generates high‐resolution data for demographic inferences (Marandel et al., 2020; O'Loughlin et al., 2014). Note also that the calculation of matrix ***R*** only requires LD information from the first temporal sample, which means the second temporal sample could be processed with lower cost technologies such as pooled sequencing, where individual genomes are pooled and sequenced together (Iranmehr et al., 2017; Schlötterer et al., 2014). Thus, cost‐effective contemporary $N_{e}$ estimation is possible through combining different sequencing technologies with the appropriate experimental designs.

We applied the method to estimate the contemporary $N_{e}$ for *A*. *coluzzii* and *A*. *gambiae* from a cluster of villages in Burkina Faso. Their genomes show great diversity, with one variant in about every two bases (Ag1000G, 2020). Although it is possible to use information from multiple chromosomes (see Appendix [Link], [Link]), chromosome 2 was excluded from demographic inferences (Ag1000G, 2020). Initially, an $\hat{N_{e}}$was provided from each of the two chromosome arms 3R and 3L separately. The estimates from both arms were very consistent with overlapping CIs. The genotypes from the two arms were then combined to provide an overall estimate per species, which was more precise. We did not observe significant differences between the estimates from ${\hat{F}}_{a}$ and ${\hat{F}}_{b}$ because the high number of loci provided sufficient drift signals. However, pseudoreplication was a severe issue here with this amount of loci. For comparison, if one naively treated all loci as independent, then the variance of $\chi_{K}^{2}$would be less than 1/6 of what we calculated from $Q^{2}$ (Figure 1). In other words, on average 6+ linked loci provided the same amount of information about genetic drift as one independent locus. Another perspective to the same question is through the “effective number of independent loci” $K^{\prime }$ given a data set (Waples, 2020), which is approximately $K^{\prime }=2K^{2}/var\left(Q^{2}\right)$. Note that $K^{\prime }$ is affected by factors such as the level of LD pruning and choice of loci, and hence can only be determined on a case‐by‐case basis.

Previous $N_{e}$ estimates for *Anopheles* mosquitos were in the order of 10^6^–10^9^ (Ag1000G, 2017; Khatri & Burt, 2019), but those were figures for the entire species rather than a local population, and also averaged over a much longer period backward in time. In contrast, our $N_{e}$ estimates are spatially and temporally restricted. Consistent with previous analyses, we assume the study population is panmictic and closed. If there is immigration into the focal population then this will affect the higher moments of $\delta_{i}$ and hence the $N_{e}$ estimates (Nunney, 2016; Wang & Whitlock, 2003). The window of *t* = 20 generations is relatively short for immigration to have any significant impact on the local $N_{e}$ estimates (Wang & Whitlock, 2003). A relevant mark–release–recapture experiment shows that the dispersal of *Anopheles* is mostly short‐range (Epopa et al., 2017), and that the migration rate per generation should be small and local. Combining the two factors, our reported estimates should reasonably represent the local and contemporary $N_{e}$. In principle, it may be possible to jointly infer $N_{e}$ and immigration rates if there are data from more than two time points or multiple geographical locations, but further theoretical work would be needed. Although Nei and Tajima (1981) comment that the effect of selection on temporal *F* is generally minor, it is a known consequence that selection affects the temporal change in allele frequency (Jónás et al., 2016). For example, mean‐reverting balancing selection dampens $var(\delta_{i})$, while with directional selection allele frequency changes are positively correlated over time. In our data analyses different measures were introduced to minimize the interference from selection. First, only single nucleotide polymorphisms (SNPs) annotated as intergenic were included. Second, the entire chromosome 2 was excluded because it contains regions with multiple segregating inversions and insecticide‐resistance alleles (Ag1000G, 2017; Ag1000G, 2020). Even though there are intergenic SNPs on chromosome 2, they are more likely to be linked with sites under selection. Third, only one SNP was chosen per 1,000‐base‐pair (bp) window to further reduce the chance of having tight linkages. While the temporal *F* uses drift signals from neutral loci, there exist other methods which were developed to incorporate selection signals (Buffalo & Coop, 2019; Khatri, 2016). Other standard assumptions for temporal methods (Waples, 1989) apply to our method as well: individuals are random samples from the population; mutation and selection are assumed to be negligible; generations are nonoverlapping; and the number of generations between samples is known without error. In our case we assumed 10 mosquito generations per year, consistent with previous studies (Ag1000G, 2017; O'Loughlin et al., 2016). However, other factors, such as the duration of wet and dry seasons, and whether the mosquitoes aestivate during the dry season (Lehmann et al., 2010), may affect this value. The estimated population size is linearly proportional to *t* (Equation 13), so that if there were only half as many generations between time points, then the estimated $N_{e}$ would be halved.

Our ${\hat{F}}_{a}$ statistic (Equation 11) follows that of Taskas and Krimbas (1971). There are alternative forms of temporal *F*, such as ${\hat{F}}_{c}$ and ${\hat{F}}_{k}$ (Waples, 1989), but they are numerically indistinguishable when loci are plentiful with reasonably large sample size and minor allele frequency (MAF) cut‐off. Our second statistic ${\hat{F}}_{b}$ is a ratio‐of‐sums statistic similar to that of Jorde and Ryman (2007). In our simulation the mean ${\hat{F}}_{a}$ and ${\hat{F}}_{b}$ were always within a few per cent from their expectations. This shows that both statistics are good estimators of the rate of loss in genetic diversity due to drift, which is at the scale of $1/2N_{e}$. The bias is unfortunately exaggerated when translated into $N_{e}$ because of the reciprocal relationship, particularly when the denominator term $\left({\hat{F}}_{a}‐\left(1/2S_{0}\right)‐\left(1/2S_{t}\right)\right)$ of Equation 12 is close to 0. Comparing the two statistics, ${\hat{F}}_{b}$ alleviated some bias in cases when the sample size to $N_{e}$ ratio was small, or more generally when there was a lack of drift signals, consistent with previous study (Jorde & Ryman, 2007). ${\hat{F}}_{b}$ differs from ${\hat{F}}_{a}$ only by the weighing scheme, as ${\hat{F}}_{a}$ is the simple arithmetic average while ${\hat{F}}_{b}$ is a weighted average of $\delta_{i}^{2}$ in which the weights are their heterozygosity. Loci at lower frequencies are given smaller weights and hence contribute less to the overall point estimate. However, there is a trade‐off between accuracy and precision, with the unequal weighting system of ${\hat{F}}_{b}$ leading to a higher variance (Jorde & Ryman, 2007). Having very few samples and a short sampling horizon may bias *F* and $N_{e}$ estimates, as investigated previously (Waples, 1989). These are intrinsic problems to the entire temporal *F* family and unfortunately our methodology is not immune to them. Excluding loci with MAF <5% is a standard safeguard to avoid extreme values in the denominator. Likelihood‐based methods may be better than the moment‐based *F* (Williamson & Slatkin, 1999), and the development of such advanced models for linked loci is worth investigating. Genetic linkage is an important factor affecting the width of CI, on top of sample sizes, *K*, $N_{e}$ and *t* (Waples, 1989), and with so many variables affecting the precision we suggest running preliminary simulations to determine appropriate sample sizes. From a practical point of view, we recommend using loci that are sparsely spread along the chromosome with lower average linkage and LD, preferably after some “LD pruning”, to lower $var(Q^{2})$. Although it is tempting to include all available loci for $N_{e}$ estimation, the marginal benefits of adding more linked loci from the same chromosome will diminish, as most information has already been captured. Instead, the excess sampling errors (on allele frequencies, pairwise LD and recombination) and computing burden may outweigh the benefits. Furthermore, it is possible to combine temporal information from more than two time points (Buffalo & Coop, 2019; Williamson & Slatkin, 1999).

The computation effort required by our method is manageable. Storing ***R*** can be memory‐hungry when many loci are involved. The calculation of all pairwise $r_{ij0}$ and $c_{ij0}$ is repetitive but parallelizable. Computing the eigenvalues of ***R*** is the most computationally demanding task, but a mainstream workstation can easily evaluate ***R*** with 33,000+ loci with an optimized linear algebra pack, as demonstrated in our worked example. The maximum number of loci a computer can handle will depend on memory.

## 6 METHODS

Computer simulations were run using fastsimcoal version 2.6 (Excoffier & Foll, 2011). Two temporally spaced genetic samples separated by *t* = 10 generations were simulated with chromosome length of 1e5 bp. The mutation rate was 1e‐6 per bp per generation, and the recombination frequency was 1e‐5 between adjacent bp per generation. Thus, the recombination rate between an arbitrary pair of loci with y bp apart is $0.5*[1‐{\left(1‐2\times 10^{‐5}\right)}^{y}]$. Different combinations of $N_{e}$, sample sizes and *K* were tested. Loci with MAF <5% at either time point were excluded. The two statistics ${\hat{F}}_{a}$ and ${\hat{F}}_{b}$ were calculated as described. While the simulator outputs phased (haplotypic) data, unphased genotypic data were mimicked by randomly pairing two haplotypes. When calculating initial $r_{ij0}$ within ***R***, slightly different treatments are required for phased and unphased data (see Appendix [Link], [Link]). In total, 10,000 realizations of $Q^{2}$ were generated from the eigenvalues of ***R*** (or $KW^{1/2}RW^{1/2}$ for ${\hat{F}}_{b}$). The 2.5‐ and 97.5‐percentiles were used to calculate the upper and lower CI of *F*. These calculations were repeated for 1,000 independent simulations for each parameter combination. Results are displayed in Table 1. Additional simulations were run to confirm the method's robustness towards different levels of genome‐wide recombination (as a ratio to chromosome length). Three recombination frequencies (1e‐4, 1e‐5 and 1e‐6 between adjacent bp per generation) with 100‐fold differences were chosen to represent cases with high, moderate and low recombination. The chromosome length for all three scenarios were set to 1e5 bp. We also ran another set of simulations to validate the method with *K* = 20,000 linked loci, a scenario comparable to our real data examples. The results from both additional simulations can be found in the Appendix [Link], [Link].

The suggested method was also applied to two real data sets of *Anopheles* mosquitoes (Ag1000G, 2017). Full details about population sampling, sequencing, accessibility, variant calling and filtering can be found in Ag1000G (2020). A brief summary of the procedure is as follows. A total of 290 *Anopheles* mosquitoes were collected from Burkina Faso at two time points in July 2012 and in July 2014. July is around the peak of the wet season and of mosquito activity. Samples were sequenced at the Wellcome Sanger Institute on the Illumina HiSeq200 platform, with targeted coverage of 30 × per individual. Sequence reads were aligned to the AgamP3 reference genome. gatk was used for SNP discovery and filtering. Furthermore, some samples were sequenced a second time using the same method in order to increase the accuracy. The samples went through several stages of quality control to remove samples of poor quality. About 6 million biallelic SNPs on chromosome arms 3R and 3L were annotated as intergenic. Only those with MAF ≥5% at both time points were included. Lastly, to avoid tightly linked SNPs, we randomly chose one SNP per 1,000‐bp window, which yielded about 15,000–18,000 SNPs per chromosome arm per species for $N_{e}$estimation. We provided $N_{e}$estimates per chromosome arm per species as well as the overall $N_{e}$estimates per species. $N_{e}$estimates calculated via ${\hat{F}}_{a}$ are reported in Table 2, while the same estimates via ${\hat{F}}_{b}$can be found in the Appendix [Link], [Link]. Data were analysed by Microsoft r open 3.5.3 (Microsoft & R. C. T., 2019; Team & R. C., 2019) .

## CONFLICT OF INTEREST

The authors declare no conflicts of interest. The funding bodies had no direct role in the design of the study nor in the collection, analysis, interpretation of data or in the writing of the manuscript.

## AUTHOR CONTRIBUTIONS

T‐Y.J.H. and A.B. conceived the research. T‐Y.J.H. ran the computer simulations and analyses. J.H.B. and T‐Y.J.H. assembled and analysed the mosquito sequences. T‐Y.J.H. wrote the manuscript with input from the other authors.

## Supporting information

Supplementary Material

Supplementary Material

## Data Availability

Mosquito data collected in 2012 belong to the Ag1000G Phase 2 release and are publicly available on the MalariaGEN website (https://www.malariagen.net/data/ag1000g‐phase‐2‐ar1). Those collected in 2014 belong to the Ag1000G Phase 3 release (https://www.malariagen.net/data/ag1000g‐phase3‐snp). The computer codes for data analyses can be found in this public Github repository (https://github.com/tinyuhui/temporal_Ne_linked). The subsets of genotypes used in the study are attached to this paper as a zip file, and are also available in the Github repository above.
