# Supplementary material for: Contemporary N e estimation using temporally spaced data with linked loci
Source: Mol Ecol Resour. 2021 Jun 22;21(7):2221–30. doi: 10.1111/1755-0998.13412 (PMC8518636; doi:10.1111/1755-0998.13412)
Supplement: Supplementary file 2 — Supplementary Material [file MEN-21-2221-s001.docx]

**Supplementary Information**

**APPENDIX S1**

Part I: Deriving the covariance of the changes in allele frequency

Let $D_{t}$ be the raw LD measure at time $t$, and $c$ be the recombination rate between a pair of loci. Hill and Robertson (1968) showed that the recurrence relation of LD is:

$$E[D_{t}]=\left( 1-\frac{1}{2N_{e}} \right)\left( 1-c \right)E[D_{t-1}]$$

$$=\ldots=\left( 1-\frac{1}{2N_{e}} \right)^{t}\left( 1-c \right)^{t}D_{0}$$

where $D_{0}$ is the (known) LD measure at time 0. Equation 1 in the main text shows the covariance of the changes in (true) allele frequency for a pair of loci. To derive it we need to consider the dynamics of the four haplotype frequencies over time. For the two-locus two-allele case, let us assume there are alleles $A$ and $a$ on the first locus, and $B$ and $b$ on the second locus. Assume we know $\underline{p_{0}}=(p_{AB0}, p_{Ab0}, p_{aB0}, p_{ab0})$, the four haplotype frequencies at the $0^{th}$ generation. The initial LD measure $D_{0}=p_{AB0}p_{ab0}-p_{Ab0}p_{aB0}$ is also known. In the next generation, the Wright-Fisher model suggests the haplotype counts follow a multinomial distribution with size $2N_{e}$, with probabilities equal the expected haplotype frequencies after recombination:

$$expected frequency for AB=p_{AB0}-cD_{0}$$

$$expected frequency for Ab=p_{Ab0}+cD_{0}$$

$$expected frequency for aB=p_{aB0}+cD_{0}$$

$$expected frequency for ab=p_{ab0}-cD_{0}$$

To obtain the haplotype frequencies we simply divide the counts by $2N_{e}$. The covariance (conditioning on $\underline{p_{0}}$) between the allele frequencies of $A$ and $B$ is $cov(p_{AB1}+p_{Ab1}, p_{AB1}+p_{aB1})$. Because of linearity, it can be broken down into four covariances:

$$cov\left( p_{AB1}+p_{Ab1}, p_{AB1}+p_{aB1} \right)=cov\left( p_{AB1}, p_{AB1} \right)+cov\left( p_{AB1}, p_{aB1} \right)+cov\left( p_{Ab1}, p_{AB1} \right)+cov(p_{Ab1},p_{aB1})$$

These four covariances are the second or product moment of multinomial distributions. For instance:

$$cov\left( p_{AB1}, p_{AB1} \right)=\frac{\left( p_{AB0}-cD_{0} \right)\left( 1-p_{AB0}+cD_{0} \right)}{2N_{e}}$$

and

$$cov\left( p_{AB1},p_{aB1} \right)=-\frac{\left( p_{AB0}-cD_{0} \right)\left( p_{aB0}+cD_{0} \right)}{2N_{e}}$$

Finally, by putting these terms together:

$$cov\left( p_{AB1}+p_{Ab1}, p_{AB1}+p_{aB1} \right)=\frac{\left( 1-c \right)D_{0}}{2N_{e}}$$

The next step is to generalise such covariance for $t$ generations ahead, that is $cov\left( p_{ABt}+p_{Abt}, p_{ABt}+p_{aBt} \right)$. At this point, we only have this covariance conditioning on $\underline{p_{t-1}}$, but ultimately we would like to obtain this covariance conditioning on $\underline{p_{0}}$. By the total law of covariance,

$$cov\left( p_{ABt}+p_{Abt}, p_{ABt}+p_{aBt} \right)=E\left[ cov\left( p_{ABt}+p_{Abt}, p_{ABt}+p_{aBt} | \underline{p_{t-1}} \right) \right]+cov[E\left( p_{ABt}+p_{Abt} | \underline{p_{t-1}} \right), E\left( p_{AB_{t}}+p_{aBt} | \underline{p_{t-1}} \right)]$$

The term inside the first expectation is $\frac{\left( 1-c \right)D_{t-1}}{2N_{e}}$ as calculated previously. Neither genetic drift nor recombination change the mean allele frequency over time, hence $E\left( p_{ABt}+p_{Abt} | \underline{p_{t-1}} \right)=p_{ABt-1}+p_{Abt-1}$. The covariance is simplified as:

$$cov\left( p_{ABt}+p_{Abt}, p_{ABt}+p_{aBt} \right)=\frac{1-c}{2N_{e}}E\left[ D_{t-1} \right]+cov(p_{ABt-1}+p_{Abt-1}, p_{ABt-1}+p_{aBt-1})$$

Now a recurrence relation is established for the covariance of the allele frequencies. The final exercise is to express $cov\left( p_{ABt}+p_{Abt}, p_{ABt}+p_{aBt} \right)$ in terms of $D_{0}$, the initial condition. It is not difficult to see that the covariance can be expressed as the weighted sum of the LD measures at different time points. In fact, it is a sum of a geometric series, with a common ratio $\left( 1-\frac{1}{2N_{e}} \right)(1-c)$:

$$cov\left( p_{ABt}+p_{Abt}, p_{ABt}+p_{aBt} \right)=\frac{\left( 1-c \right)D_{0}}{2N}\left\{ \left( 1-\frac{1}{2N_{e}} \right)^{t-1}\left( 1-c \right)^{t-1}+\left( 1-\frac{1}{2N_{e}} \right)^{t-2}\left( 1-c \right)^{t-2}+\ldots+\left( 1-\frac{1}{2N_{e}} \right)^{1}\left( 1-c \right)^{1}+1 \right\}$$

$$=\frac{\left( 1-c \right)[1-\left( 1-\frac{1}{2N_{e}} \right)^{t}\left( 1-c \right)^{t}]}{2N_{e}[1-\left( 1-\frac{1}{2N_{e}} \right)\left( 1-c \right)]}D_{0}$$

This completes the derivation of Equation 1 in the main text. Note that all equations above are conditional on $\underline{p_{0}}$, hence the covariance of allele frequencies is identical to the covariance of the changes in allele frequency.

Part II: Distributions of $Q^{2}$

In a relatively short time frame $t$, the standardised temporal changes in observed frequency $\underline{\delta}=\left( \delta_{1},\ldots,\delta_{K} \right)^{T}$ on $K$ different loci can be modelled by a multivariate normal distribution, with mean vector zero and a covariance matrix. We can decorrelate $\underline{\delta}$ via eigenvalue decomposition of the covariance matrix. Since all $\delta_{i}$ share a common variance (main text Eqn 7 and 9), working on the correlation matrix $\boldsymbol{R}$ is just as good.

Let $\boldsymbol{P}$ be the matrix holding all the eigenvectors of the covariance matrix, and $\underline{z}=\left( z_{i},\ldots,z_{K} \right)^{T}$ be the transformed (independent) multivariate normal variables. Note that $\boldsymbol{P}^{\boldsymbol{T}}\boldsymbol{P}=\boldsymbol{P}\boldsymbol{P}^{\boldsymbol{T}}=\boldsymbol{I}$ and $\underline{z}=\boldsymbol{P}^{\boldsymbol{T}}\underline{\delta}$. Each $z_{i}\sim N(0,\lambda_{i})$ independently, where $\lambda_{i}$ is the i-th eigenvalue.

Note that the statistic $\hat{F}_{a}\propto\sum\delta_{i}^{2} =\underline{\delta}^{T}\underline{\delta}=\left( \boldsymbol{P}\underline{z} \right)^{T}\left( \boldsymbol{P}\underline{z} \right)=\underline{z}^{T}\boldsymbol{P}^{\boldsymbol{T}}\boldsymbol{P}\underline{z}=\underline{z}^{T}\underline{z}=\sum z_{i}^{2}$. Thus, that the distribution of $\hat{F}_{a}$ is proportional to the sum of $K$ independent squared normal variables $z_{i}^{2}$, which can be easily generated by computer. For the same reason, $K\hat{F}_{a}/F$, the scaled measure (main text Eqn 15), is distributed as $Q^{2}$, the sum of $K$ independent squared normal variables, whose variances are the eigenvalues of the correlation matrix $\boldsymbol{R}$. For $\hat{F}_{b}$, $\hat{F}_{b}=\sum w_{i}\delta_{i}^{2}=\left( \boldsymbol{W}^{\frac{\boldsymbol{1}}{\boldsymbol{2}}}\underline{\delta} \right)^{T}\left( \boldsymbol{W}^{\frac{\boldsymbol{1}}{\boldsymbol{2}}}\underline{\delta} \right)\boldsymbol{=}\underline{\delta}^{T}\boldsymbol{W}\underline{\delta}$. Because of the unequal weights being assigned to each $\delta_{i}^{2}$, the eigenvalues of $K\boldsymbol{W}^{\boldsymbol{1/2}}\boldsymbol{R}\boldsymbol{W}^{\boldsymbol{1/2}}$ are considered when generating the $Q^{2}$ distribution.

When calculating the eigenvalues, we have had cases with very small negative eigenvalues, which should not have happened to a positive-definite matrix. This might be caused by the accumulation of estimation and rounding errors. While this effect was very minor, we imposed a cut-off (say, 1e-7) to remove eigenvalues that were smaller than this value. These linear combinations of SNPs associated with small eigenvalues provide very little additional information on $N_{e}$, as information has already been reflected by those with larger eigenvalues.

Part III: Additional simulation results

Additional simulations were run to investigate the method’s robustness towards different genome-wide recombination frequencies. Three recombination frequencies (1e-4, 1e-5, and 1e-6, between adjacent bp per generation) were tested to represent cases of high, medium, and low recombination. Across all three cases, the same chromosome length of 1e5 bp and mutation rate of 1e-6 (per bp per generation) were used. The true $N_{e}$ was 5,000 with 2,000 loci. The sample sizes were 100 individuals each at two time points of $t=10$ generations apart. The results, based on 1,000 independent simulations, are shown below:

| Recombination frequency | $mean\left( \hat{F}_{a} \right)$ & corresponding $\hat{N_{e}}$ | $SD(\hat{F}_{a})$ | $\hat{F}_{a}$ Adjusted 95% C.I. coverage (phased data) | $mean\left( \hat{F}_{b} \right)$ & corresponding $\hat{N_{e}}$ | $SD(\hat{F}_{b})$ | $\hat{F}_{b}$ Adjusted 95% C.I. coverage (phased data) | Unadjusted 95% C.I. coverage (assumed independence) |
| --- | --- | --- | --- | --- | --- | --- | --- |
| 1e-4 (high) | 0.01062 (8,456) | 3.883e-4 | 0.977 | 0.01079 (6,359) | 4.149e-4 | 0.980 | 0.832 |
| 1e-5 (medium) | 0.01063 (7,949) | 4.97e-4 | 0.962 | 0.01079 (6,344) | 5.222e-4 | 0.966 | 0.801 |
| 1e-6 (low) | 0.01059 (8,456) | 1.209e-3 | 0.945 | 0.01075 (6,688) | 1.272e-3 | 0.948 | 0.403 |

Under high recombination rate loci were mostly loosely linked, hence the unadjusted C.I. had higher coverage, but were still far below the targeted ratio of 95%. Severe pseudo-replication occurred at low recombination frequency with the highest standard deviation. Our adjusted C.I. achieved the targeted coverage in all three cases.

Another set of simulations was run to evaluate the method’s performance with $K=20,000$ linked loci, a number comparable to our real datasets. The $N_{e}$ was 10,000 throughout the sampling horizon. The sample sizes were 100 individuals each at two time points of $t=10$ generations apart. Other parameters, such as chromosome length, recombination and mutation rates, were the same as in Table 1 from the main text. The only difference was that a larger historical $N_{e}$ of 50,000 was simulated in order to generate polymorphic SNPs of this number. The population size has shrunk back to the targeted contemporary $N_{e}$ of 10,000 just before sampling. The results, based on 1,000 independent simulations, can be found below:

| $mean\left( \hat{F}_{a} \right)$ & corresponding $\hat{N_{e}}$ | $SD(\hat{F}_{a})$ | $\hat{F}_{a}$ Adjusted 95% C.I. coverage (phased data) | $mean\left( \hat{F}_{b} \right)$ & corresponding $\hat{N_{e}}$ | $SD(\hat{F}_{b})$ | $\hat{F}_{b}$ Adjusted 95% C.I. coverage (phased data) | Unadjusted 95% C.I. coverage (assumed independence) |
| --- | --- | --- | --- | --- | --- | --- |
| 0.01016 (31,449) | 2.228e-4 | 0.979 | 0.01031 (16,587) | 2.298e-4 | 0.981 | 0.656 |

The proportions of C.I. covering $mean(\hat{F}$) for the two statistics reached the targeted level under $K=20,000$ loci. They were slightly conservative but still performed much better than the unadjusted C.I.. There are two extra implications from this simulation: 1) Population sizes outside the sampling horizon do not affect the contemporary $N_{e}$ point estimates. 2) The high historical $N_{e}$ reduces the average pairwise $r_{ij0}$, therefore increases the unadjusted C.I. coverage. This explains why unadjusted C.I. coverage here was higher than the last row from Table 1 in the main text, despite using more tightly linked loci (20,000 vs 5,000).

Part IV: Estimating $N_{e}$ for *An. coluzzii* and *An. gambiae*

In the main text we described how the data was collected, sequenced, and filtered. The sample sizes, number of loci, and $\hat{N_{e}}$ are shown in main text Table 2. The two-step procedure below converts the physical distance (in bp) to recombination rate $c$ for a given pair of loci. First we calculate the genetic distance $d$ in Morgans:

$$d=\left( physical distance in megabase \right)*\frac{1.4}{100}$$

This is based on the results by Pombi et al. (2006) that recombination occurs at about 1.4 centimorgan per megabase (cM/Mb) on chromosome 3 of *An. coluzzii* and *An. gambiae*. The next step is to convert $d$ into $c$ via the Haldane mapping function:

$$c=0.5*(1-e^{-2d})$$

Note that the Haldane mapping function guarantees that recombination rate $c$ is bounded between 0 and 0.5. With all the input parameters, we computed the eigenvalues of $\boldsymbol{R}$, and then generated the distribution of $Q^{2}$ with 50,000 realisations. The C.I. for $F$ and $N_{e}$ can be obtained from the empirical quantiles of $Q^{2}$ as described in the main text.

| Species | Chrom | $S_{0}$ | $S_{t}$ | $K$ | $\hat{F}_{b}$ | $\hat{N_{e}}$ | Combined $\hat{N_{e}}$ |
| --- | --- | --- | --- | --- | --- | --- | --- |
| *An. coluzzii* | 3R | 82 | 53 | 17,837 | 0.0169175 | 7215 [4,294 - 22,040] | 7,563 [4,798 - 18,171] |
| *An. coluzzii* | 3L | 82 | 53 | 15,317 | 0.0167809 | 8004 [4,383 - 39,713] |  |
| *An. gambiae* | 3R | 92 | 45 | 17,963 | 0.0180633 | 6590 [3,965 - 17,944] | 6,057 [4,086 - 11,557] |
| *An. gambiae* | 3L | 92 | 45 | 15,409 | 0.0183505 | 5441 [3,432 - 13,832] |  |

Table 2 (main text) reports the $N_{e}$ estimates via $\hat{F}_{a}$. The same analysis with $\hat{F}_{b}$ can be found in the table below. Note that the point and C.I. estimates from both statistics were very similar.

Part V: Combining $N_{e}$ estimates from multiple chromosomes

It is possible to combine genotypic information from two or more chromosomes to provide an overall $\hat{N_{e}}$ estimate. Let us assume we have obtained $\hat{F}_{a1}$ and $\hat{F}_{a2}$ from two chromosomes of the same population. Let $K_{1}$ and $K_{2}$ be their numbers of (linked) loci, and $\boldsymbol{R}_{\boldsymbol{1}}$ and $\boldsymbol{R}_{\boldsymbol{2}}$ be their correlation matrices. The overall point estimate $\hat{F}_{all}$ is the average of all standardised changes in observed frequency, which is also the weighted average of individual $\hat{F}_{a}$’s:

$$\hat{F}_{all}=\frac{K_{1}\hat{F}_{a1}+K_{2}\hat{F}_{a2}}{K_{1}+K_{2}}$$

[SI4]

Let $\underline{\lambda_{1}}$ be a vector (with length $K_{1}$) of eigenvalues of $\boldsymbol{R}_{\boldsymbol{1}}$, and $\underline{\lambda_{2}}$ (with length $K_{2}$) be that of $\boldsymbol{R}_{\boldsymbol{2}}$. If we assume loci on different chromosomes are independent, and that their correlation in the changes in observed frequency is zero, then the combined correlation matrix $\boldsymbol{R}_{\boldsymbol{all}}$ can be expressed as a block matrix:

$$\boldsymbol{R}_{\boldsymbol{all}}=\left[ \begin{matrix} \boldsymbol{R}_{\boldsymbol{1}} & \boldsymbol{0} \\ \boldsymbol{0} & \boldsymbol{R}_{\boldsymbol{2}} \end{matrix} \right]$$

[SI5]

Note that $\boldsymbol{R}_{\boldsymbol{all}}$ has $(K_{1}+K_{2})$ rows and columns, and $\boldsymbol{0}$ is a zero matrix. In practice there is no need to find the eigenvalues of $\boldsymbol{R}_{\boldsymbol{all}}$ again, because they are simply the eigenvalues of $\boldsymbol{R}_{\boldsymbol{1}}$ and $\boldsymbol{R}_{\boldsymbol{2}}$ combined:

$$\underline{\lambda_{all}}=(\underline{\lambda_{1}}, \underline{\lambda_{2}})$$

[SI6]

With $\hat{F}_{all}$ and $\underline{\lambda_{all}}$ we can generate the $Q^{2}$ distribution for the combined dataset. The idea can easily be generalised to combine $\hat{F}$’s from an arbitrary number of chromosomes (though note that, due to shared underlying pedigree, unlinked loci are not strictly independent, as discussed in King et al. (2017)).

Part VI: $Q^{2}$ and the mis-estimation of $\hat{N_{e}}$

In the main text we mentioned the distribution of $Q^{2}$ is insensitive to the mis-estimation of $\hat{N_{e}}$, that even a 10-fold over- or under-estimation of $N_{e}$ will not severely affect its shape and variance. We demonstrate this property using the results from chromosome 3R of *An. gambiae* (main text Table 2, $\hat{N_{e}}=5,532$ with 17,963 loci). After obtaining $\hat{N_{e}}$, we re-compute the correlation matrix $\boldsymbol{R}$ and its eigenvalues by artificially setting $N_{e}=0.1\hat{N_{e}}$ or $10\hat{N_{e}}$ (via Eqn 10, while keeping all other parameters $r_{ij0},c_{ij}, t, S_{0}, S_{t}$ the same). With three different $\boldsymbol{R}$, we can generation three different $Q^{2}$ distributions. The three $Q^{2}$ are plotted side by side below, with the original $Q^{2}$ at the middle:


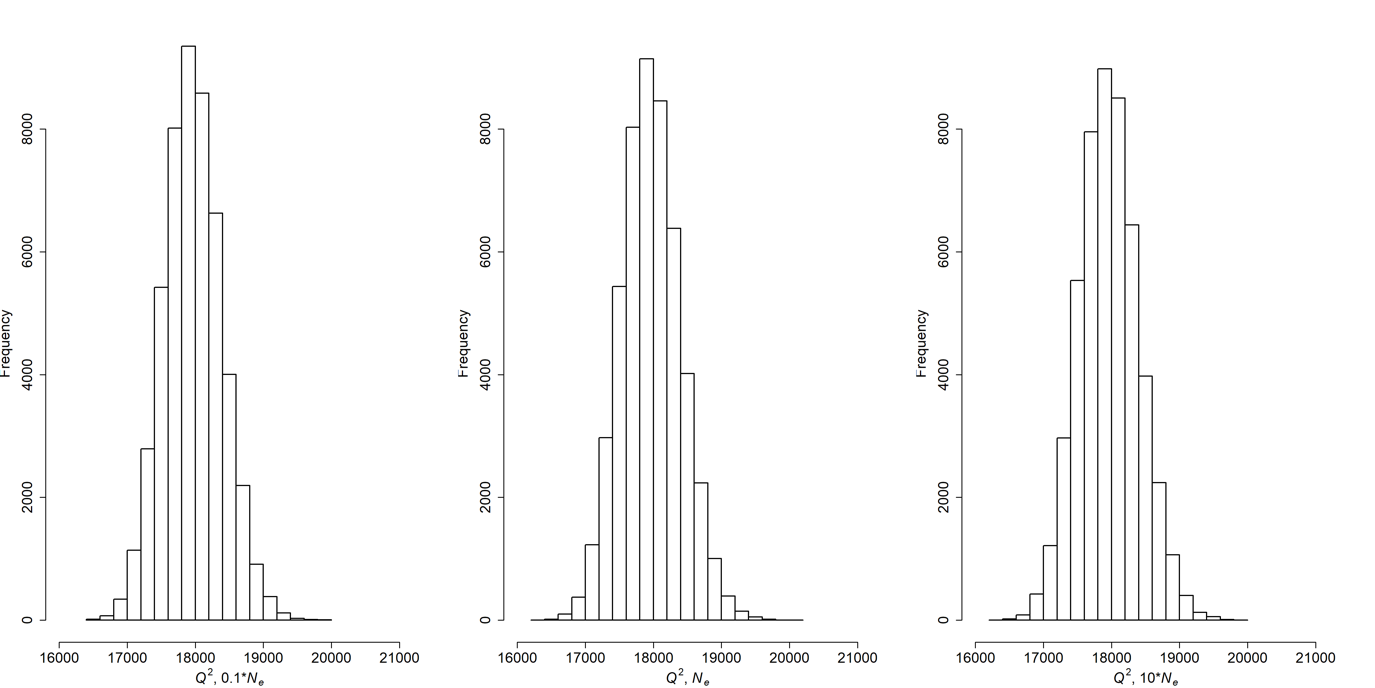


The three plots are visually indistinguishable. When comparing their variances, at $0.1\hat{N_{e}}$ the difference is +0.98%, and at $10\hat{N_{e}}$ it is -0.59%.

Part VII: Estimating pairwise $r_{ij0}$

Pairwise $r_{ij0}$ is a key component in the correlation matrix $\boldsymbol{R}$ (main text Equation 10). For a pair of biallelic loci, let $(p_{AB0},p_{Ab0},p_{aB0},p_{ab0})$ be the initial haplotype frequencies, then

$$r=\frac{p_{AB0}-p_{ab0}}{\sqrt{(p_{AB0}+p_{Ab0})(p_{aB0}+p_{ab0})(p_{AB0}+p_{aB0})(p_{Ab0}+p_{ab0})}}$$

This quantity is usually not known and needs to be estimated from the data. During our development we found that using the MLE for $r_{ij0}$ will overestimate its magnitude and hence the variance of $Q^{2}$. This results in the C.I. being slightly more conservative than it should be. Although it does little harm in practice, we aim to further correct it for better and tighter C.I. coverage.

We also found that different treatments are required for phased and unphased data. For phased data, the MLE for the four haplotype frequencies are simply their relative proportions in the samples. We can use these four estimates to compute $\hat{r}$ and $\hat{r^{2}}$, the MLE for the LD coefficients. While the sample size correction for $\hat{r}$ is not known, the correction for $\hat{r^{2}}$ is $(\hat{r^{2}}-\frac{1}{{2S}_{0}})/(1-\frac{1}{{2S}_{0}})$ (Hui and Burt, 2020), where $S_{0}$ is the diploid sample size for the first temporal sample. We then approximate $\hat{r}_{ijo}$ by combining $\hat{r}$ and the square root of the corrected $\hat{r^{2}}$:

$$\hat{r}_{ijo}\approx\frac{1}{2}[\hat{r}+\left( sign of \hat{r} \right)*\sqrt{\max\left( \frac{\hat{r^{2}}-\frac{1}{{2S}_{0}}}{1-\frac{1}{{2S}_{0}}}, 0 \right)}]$$

We implemented this calculation in our computer simulations (main text Table 1).

When gametic phase is unknown (i.e. only genotypic data is available), the MLE for the four haplotype frequencies can be estimated by the EM algorithm or other equivalent methods (Excoffier and Slatkin, 1995; Hui and Burt, 2020). $\hat{r}$ and $\hat{r^{2}}$ are computed in the same way, but naturally they have larger variances than their phased counterparts. Hence a stronger correction can be afforded:

$$\hat{r}_{ijo}\approx(sign of \hat{r})*\sqrt{\max\left( \frac{\hat{r^{2}}-\frac{1}{S_{0}}}{1-\frac{1}{S_{0}}}, 0 \right)}]$$

We implemented this calculation in our computer simulations (main text Table 1, SI Part II) and in the real data examples for *An. coluzzii* and *An. gambiae*.

References

Excoffier, L., and M. Slatkin, 1995 Maximum-likelihood estimation of molecular haplotype frequencies in a diploid population. Mol. Biol. Evol. **12:**921-927.

Hill, W. G., and A. Robertson, 1968 Linkage disequilibrium in finite populations. TAG.Theoretical and applied genetics.Theoretische und angewandte Genetik **38:**226-231.

Hui, T. J., and A. Burt, 2020 Estimating linkage disequilibrium from genotypes under hardy-weinberg equilibrium. BMC genetics **21:**1-11.

King, L., J. Wakeley and S. Carmi, 2018 A non-zero variance of Tajima’s estimator for two sequences even for infinitely many unlinked loci. Theor. Popul. Biol. **122:**22-29.

Pombi, M., A. D. Stump, A. Della Torre and N. J. Besansky, 2006 Variation in recombination rate across the X chromosome of anopheles gambiae. Am. J. Trop. Med. Hyg. **75:**901-903.
